# Supplementary material for: Statin-mediated disruption of Rho GTPase prenylation and activity inhibits respiratory syncytial virus infection
Source: Commun Biol. 2021 Oct 29;4:1239. doi: 10.1038/s42003-021-02754-2 (PMC8556396; doi:10.1038/s42003-021-02754-2)

Supplementary information

Statin-mediated disruption of Rho GTPase prenylation and activity inhibits respiratory syncytial virus infection

Manpreet Malhi[1,2], Michael J. Norris[3,4], Wenming Duan[4], Theo J. Moraes[3,4,5], Jason T. Maynes[1,2,6]

[1]Department of Biochemistry, University of Toronto, Toronto, Ontario, Canada; [2]Program in Molecular Medicine, The Hospital for Sick Children, Toronto, Ontario, Canada; [3]Department of Laboratory Medicine and Pathobiology, University of Toronto, Toronto, Ontario, Canada; [4]Program in Translational Medicine, The Hospital for Sick Children, Toronto, Ontario, Canada; [5]Department of Paediatrics, Division of Respiratory Medicine, The Hospital for Sick Children, Toronto, Ontario, Canada; [6]Department of Anesthesia and Pain Medicine, The Hospital for Sick Children, Toronto, Ontario, Canada

Supplementary Table 1. Complete list of hits identified after screening for RSV inhibitors. HEp-2 cells were infected with RSV-GFP (MOI 0.5) and treated 2 hours post-infection with 2,400 compounds from the MicroSource SPECTRUM Collection at 10.0 µM. Automated image analysis was performed in CellProfiler to determine RSV-GFP inhibition (%) (see Figure 1, Methods).

Compound name	Molecular formula	RSV-GFP inhibition (%)
Emetine dihydrochloride	C29H42Cl2N2O4	100
Ouabain	C29H44O12	100
Lanatoside C	C49H76O20	100
Digoxin	C41H64O14	99.0761037
Digitoxin	C41H64O13	98.8417666
Mycophenolic acid	C17H20O6	98.7724233
Mycophenolate mofetil	C23H31NO7	98.4014151
Peruvoside	C30H44O9	98.1868948
Salinomycin, sodium	C42H69NaO11	96.4478276
Monensin A	C37H63NaO10	93.9260335
Niclosamide	C13H8Cl2N2O4	93.8508709
Gitoxigenin diacetate	C27H38O7	91.7599489
Strophanthidin	C23H32O6	90.410301
Dihydrocelastryl diacetate	C33H44O6	89.9640356
Oxaliplatin	C8H14N2O4Pt	88.7008574
Valinomycin	C54H90N6O18	88.0261719
Gentian violet	C25H30ClN3	86.3377916
Cyclopamine	C27H41NO2	86.0171837
Gramicidin A	C99H140N20O17	85.7448803
Gitoxin	C41H64O14	85.6815325
Digoxigenin	C23H34O5	85.0758283
Testosterone propionate	C22H32O3	84.7170732
Hexachlorophene	C13H6Cl6O2	83.1599419
Dihydrocelastrol	C29H40O4	82.5801727
Strophanthidinic acid lactone acetate	C25H32O7	82.3305362
Proscillaridin	C29H40O9	81.6124324
Tyrothricin	C66H85N11O15	79.9484074
Antimycin A1	C27H38N2O9	79.4899939
Benzalkonium chloride	C22H40ClN	77.3377037
Norethynodrel	C20H26O2	75.3645289
6,4’-Dihydroxyflavone	C15H10O4	75.2809702
Norethindrone	C20H26O2	75.237501
Cycloheximide	C15H23NO4	74.9912712
6-Hydroxyflavone	C15H10O3	74.1388665
Pristimerin	C30H40O4	74.085736
Fluvastatin	C24H26FNO4	73.2172555
Colforsin	C22H34O7	72.4856698
Cetrimonium bromide	C19H42BrN	71.2495374
Estradiol	C18H24O2	70.6759535
7-Desacetoxy-6,7-dehydrogedunin	C26H30O5	69.3844729
Nonoxynol-9	C33H60O10	69.2352638
Chlormidazole	C15H13ClN2	68.5336257
Drospirenone	C24H30O3	68.3325283
Methylene blue	C16H20ClN3OS	67.8195113
Alexidine hydrochloride	C26H58Cl2N10	66.8158876
Simvastatin	C25H38O5	64.3275252
Norgestrel	C21H28O2	64.1402868
Dequalinium chloride	C30H40Cl2N4	63.8321576
Alpha-toxicarol	C23H22O7	63.5771239
Pitavastatin calcium	C25H23CaFNO4	63.3720251
Pentamidine isethionate	C23H36N4O10S2	60.2369415
4’-Methoxyflavone	C16H12O3	59.909948
Aminacrine	C13H10N2	58.2834817
Atorvastatin calcium	C33H33CaFNO5	56.4280727
Cetylpyridinium chloride	C21H38ClN	55.8179833
Camptothecin	C20H16N2O4	55.7259072
Irinotecan hydrochloride	C33H39ClN4O6	55.5586307
Norgestimate	C23H31NO3	55.0066198
Norethindrone acetate	C22H28O3	54.1684635
Progesterone	C21H30O2	50.4661536

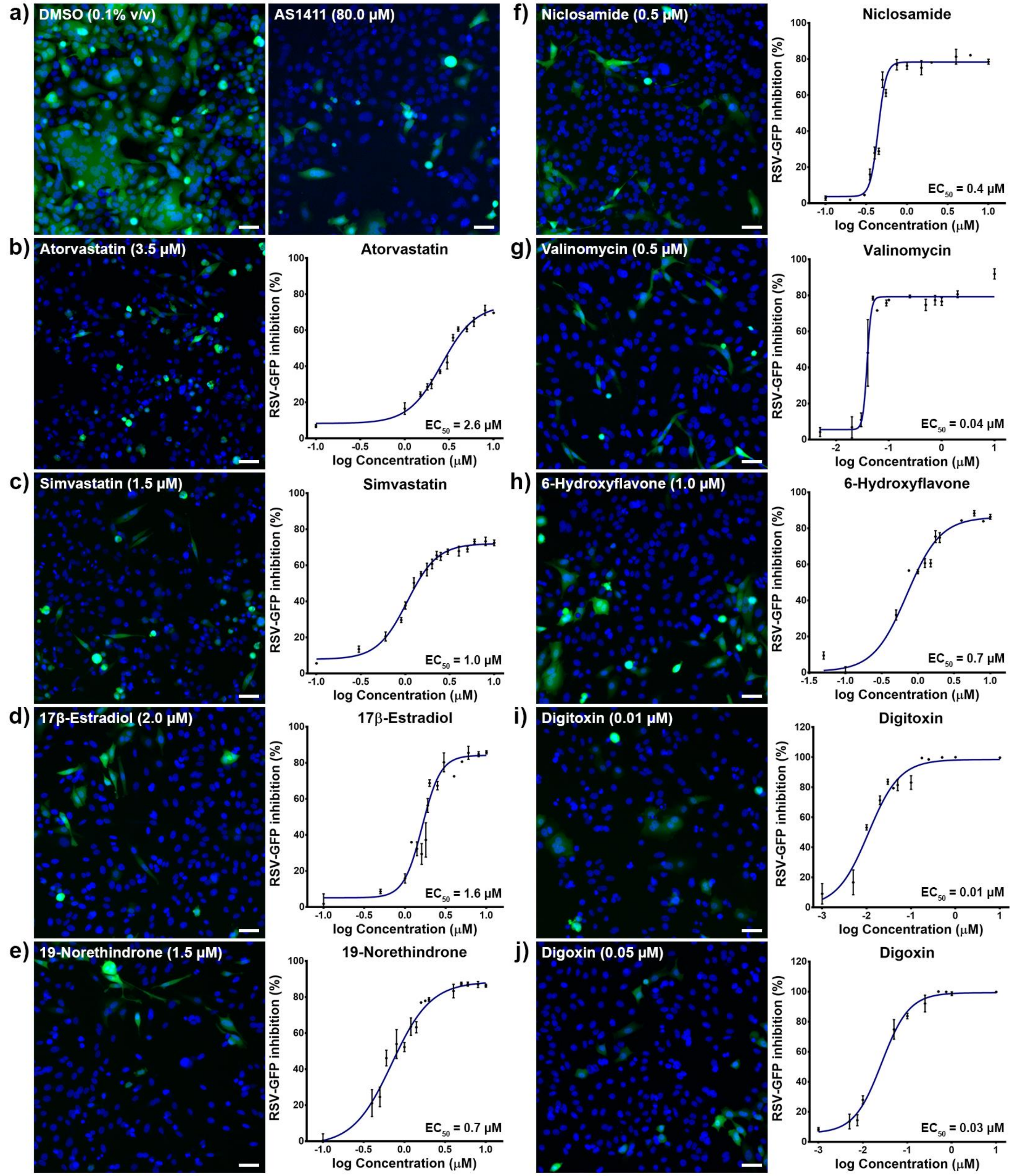

Supplementary Figure 1. Dose-dependent inhibition of RSV in HEp-2 cells by screening hits representative of each identified drug class. Representative images of HEp-2 cells infected with RSV-GFP (MOI 0.5) and treated with selected screening hits 2 hours post-viral exposure. Dose response curves corresponding to the drugs are shown to the right of each fluorescent image. Cells were treated with **(a)** DMSO (0.1% v/v) or AS1411 (80.0 μM), **(b)** atorvastatin (3.5 μM), **(c)** simvastatin (1.5 μM), **(d)** 17β-estradiol (2.0 μM), **(e)** 19-norethindrone (1.5 μM), **(f)** niclosamide (0.5 μM), **(g)** valinomycin (0.5 μM), **(h)** 6-hydroxyflavone (1.0 μM), **(i)** digitoxin (0.01 μM), or **(j)** digoxin (0.05 μM). Scale bars = 100 μm. Values are reported as means ± SEM (*n* ≥ 2 independent measurements).

Supplementary Table 2. Complete list of unique protein targets for compound hits identified from the RSV inhibitor screen. To determine requisite host pathways for RSV during infection, protein targets corresponding to each drug screen hit were identified using DrugBank. Out of 60 drug screen hits, 31 compounds had known protein targets which generated a final list of 133 unique proteins.

List of unique protein targets for drug screen hits (UniProt IDs)		
P08183	O14521	P23141
P05023	Q92887	O94956
P11387	P05177	P00441
Q9Y6L6	O95718	P02763
O95342	Q8TCC7	P20701
P06149	P63092	P46059
P06401	P04278	P09211
P35354	P22309	P04035
P0A3R9	O14717	Q86UW1
P22303	P27487	Q02928
P03372	P05093	P04731
P12268	O15431	Q9HB55
P04035	P10632	Q86UW2
P10275	P98170	Q9HAW9
Q79FX6	P35503	P12104
P33402	P35354	P02795
Q969P6	Q16678	P50993
P0A0N5	Q6ZQN7	O15439
P02768	O95622	P09488
P08183	Q9HAW7	P33527
Q9NPD5	P10635	O15440
P00367	Q92731	P15559
P03372	O75751	P35670
P08235	P20815	O00748
P11712	O60656	Q04656
P11474	P29973	P06276
P00722	P27169	Q5T3U5
P46721	P13569	P35869
Q08462	O95342	Q03393
P10275	P19224	Q9NSA0
P05108	P04798	Q92769
P20839	P31213	P11509
P33261	P11511	Q6IPK9
O75469	Q12791	P41145
P08684	P04150	Q14994
P21964	Q9NYB5	Q96BD0
P9WPB7	Q9H8P0	P43681
P29475	P30711	Q15596
P18405	Q14973	Q99527
O15245	P13637	P00846
O15244	Q9Y6L6	Q14457
P11387	Q9HAW8	P37059
P31224	P05164	P62508
Q9UNQ0	P20813	
P16662	P24462	

Supplementary Table 3. GO biological pathway and cellular component enrichment analysis on the known protein targets of drug screen hits. Characterized molecular targets of the drug screen hits were compiled into a list of 133 unique proteins (see Supplementary Table 2) and queried in PANTHER to determine functionally enriched biological pathways and cellular components. See Figure 1e, 1f for visual interactome analyses.

GO Term	GO Biological Process	Count in gene set	False discovery rate
GO:0071466	Cellular response to xenobiotic stimulus	29	3.93E-30
GO:0006805	Xenobiotic metabolic process	26	8.94E-30
GO:0042493	Response to drug	46	7.42E-28
**GO:0008202**	**Steroid metabolic process**	**29**	**1.21E-25**
GO:0042221	Response to chemical	77	5.45E-23
GO:0070887	Cellular response to chemical stimulus	62	1.66E-21
GO:0010817	Regulation of hormone levels	32	6.13E-21
**GO:0033993**	**Response to lipid**	**37**	**5.81E-20**
GO:0044281	Small molecule metabolic process	50	1.08E-19
GO:0014070	Response to organic cyclic compound	37	3.03E-19
GO:0071407	Cellular response to organic cyclic compound	29	6.48E-18
GO:0017144	Drug metabolic process	31	1.19E-17
GO:0006082	Organic acid metabolic process	36	4.48E-17
GO:0034754	Cellular hormone metabolic process	17	1.63E-16
**GO:0006629**	**Lipid metabolic process**	**38**	**6.34E-16**

GO Term	GO Cellular Component	Count in gene set	False discovery rate
GO:0005789	Endoplasmic reticulum membrane	35	2.60E-15
GO:0044432	Endoplasmic reticulum part	39	2.60E-15
GO:0098827	Endoplasmic reticulum subcompartment	35	2.60E-15
GO:0005783	Endoplasmic reticulum	42	1.32E-13
GO:0031984	Organelle subcompartment	38	3.09E-12
GO:0044425	Membrane part	78	3.09E-12
GO:0098590	Plasma membrane region	28	8.83E-10
GO:0044459	Plasma membrane part	44	1.68E-09
GO:0005887	Integral component of plasma membrane	33	2.60E-09
GO:0016020	Membrane	84	2.60E-09

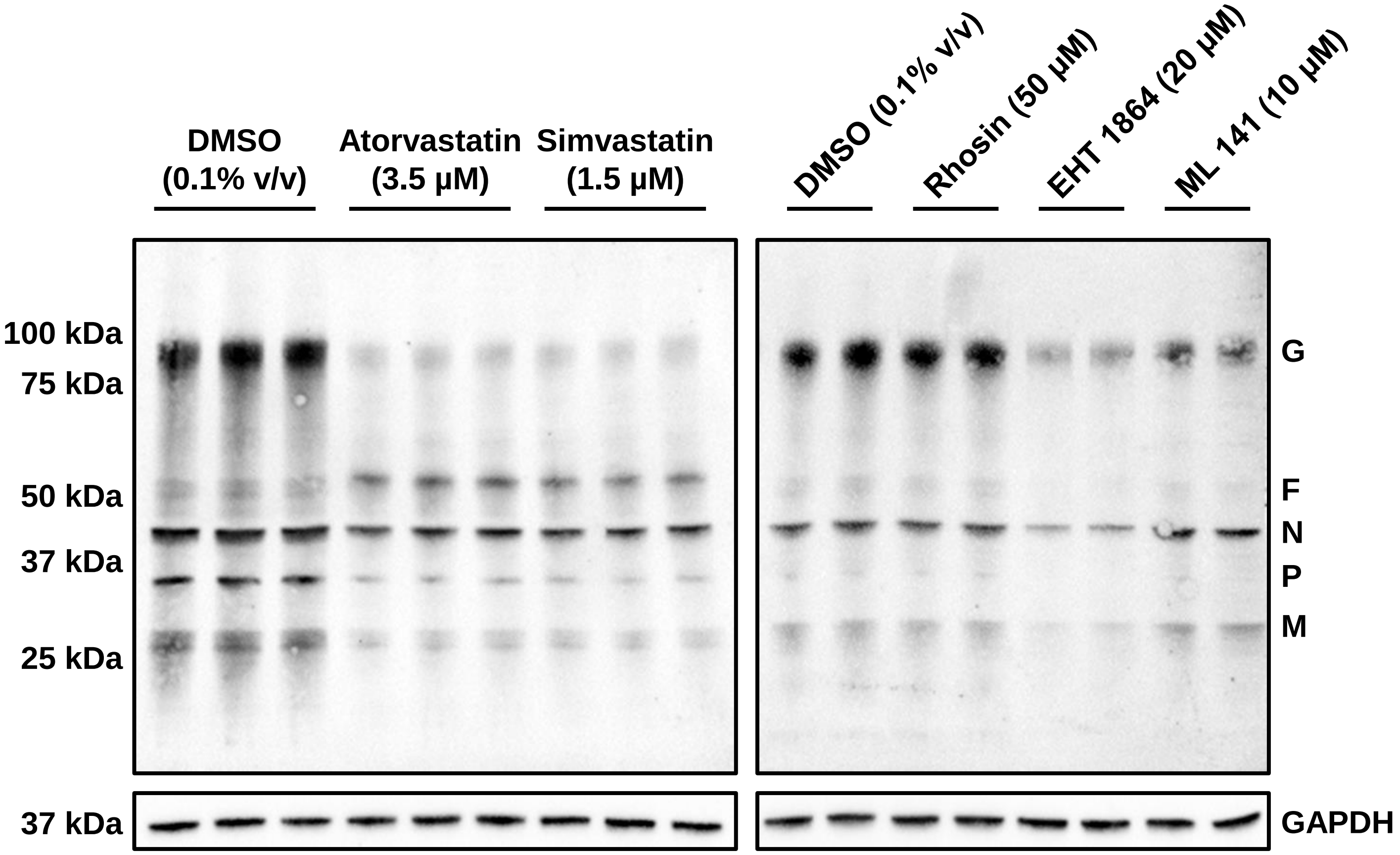

Supplementary Figure 2. Changes to viral protein expression by statin or Rho GTPase inhibitor treatment. HEp-2 cells were infected with RSV A2 (MOI 0.5) and treated with atorvastatin (3.5 µM), simvastatin (1.5 µM), Rhosin (50 µM), EHT 1864 (20 µM), or ML 141 (10 µM) 2 hours post-viral exposure. Cell lysates were collected 24 h p.i. and viral protein expression was probed using a pan-RSV polyclonal antibody (B65860G) which detects all viral antigens (n ≥ 2 cell lysates). GAPDH controls were processed on parallel blots with the same samples and protein load.

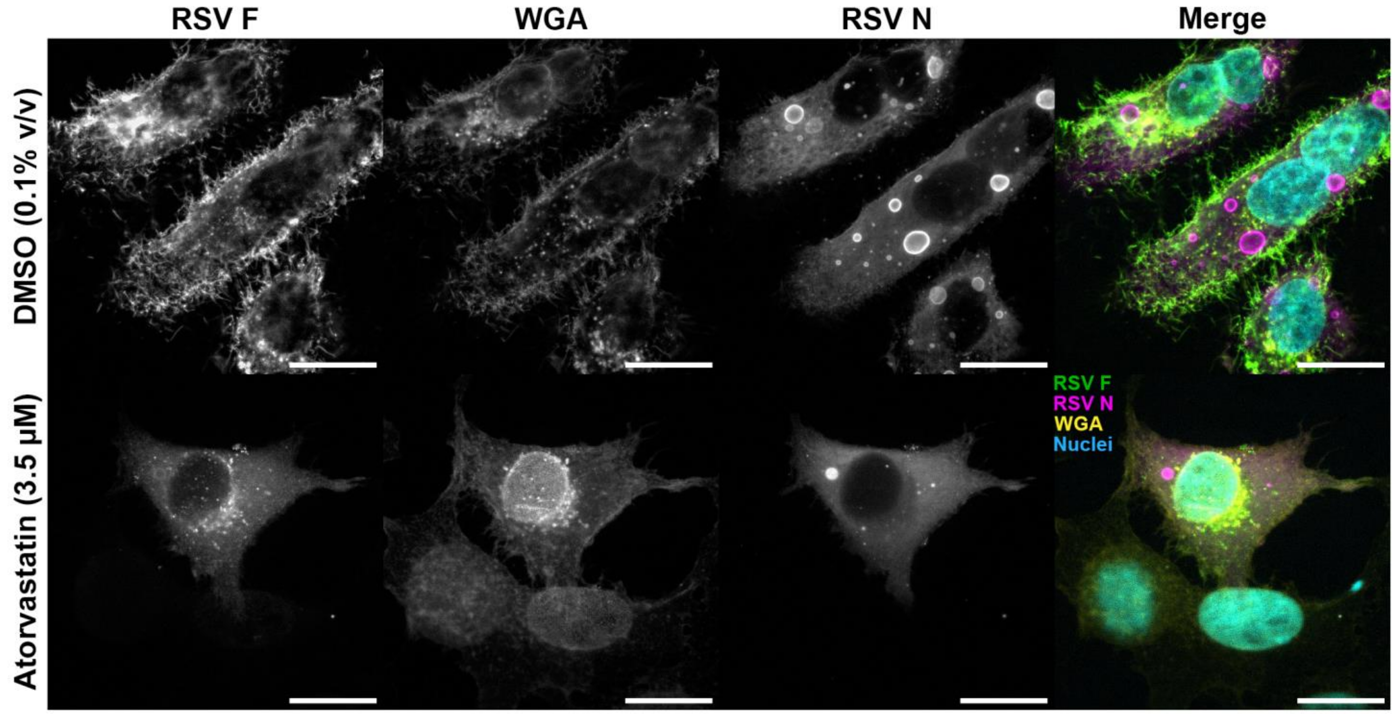

Supplementary Figure 3. Co-staining of the plasma membrane and viral proteins. HEp-2 cells were infected with RSV A2 (MOI 0.5) and visualized by high resolution confocal microscopy 48 h p.i. **(A)** Wheat germ agglutinin (WGA) was used to stain for the plasma membrane (yellow). Samples were also co-stained for the RSV F (green) and N (magenta) proteins. Atorvastatin (3.5 µM) treatment causes a reduction in the surface expression of the F protein, relative to cells treated with DMSO control (0.1% v/v). It should be noted that WGA also stains glycoproteins, therefore there is overlap in signal with the F protein. Scale bars = 20 µm.

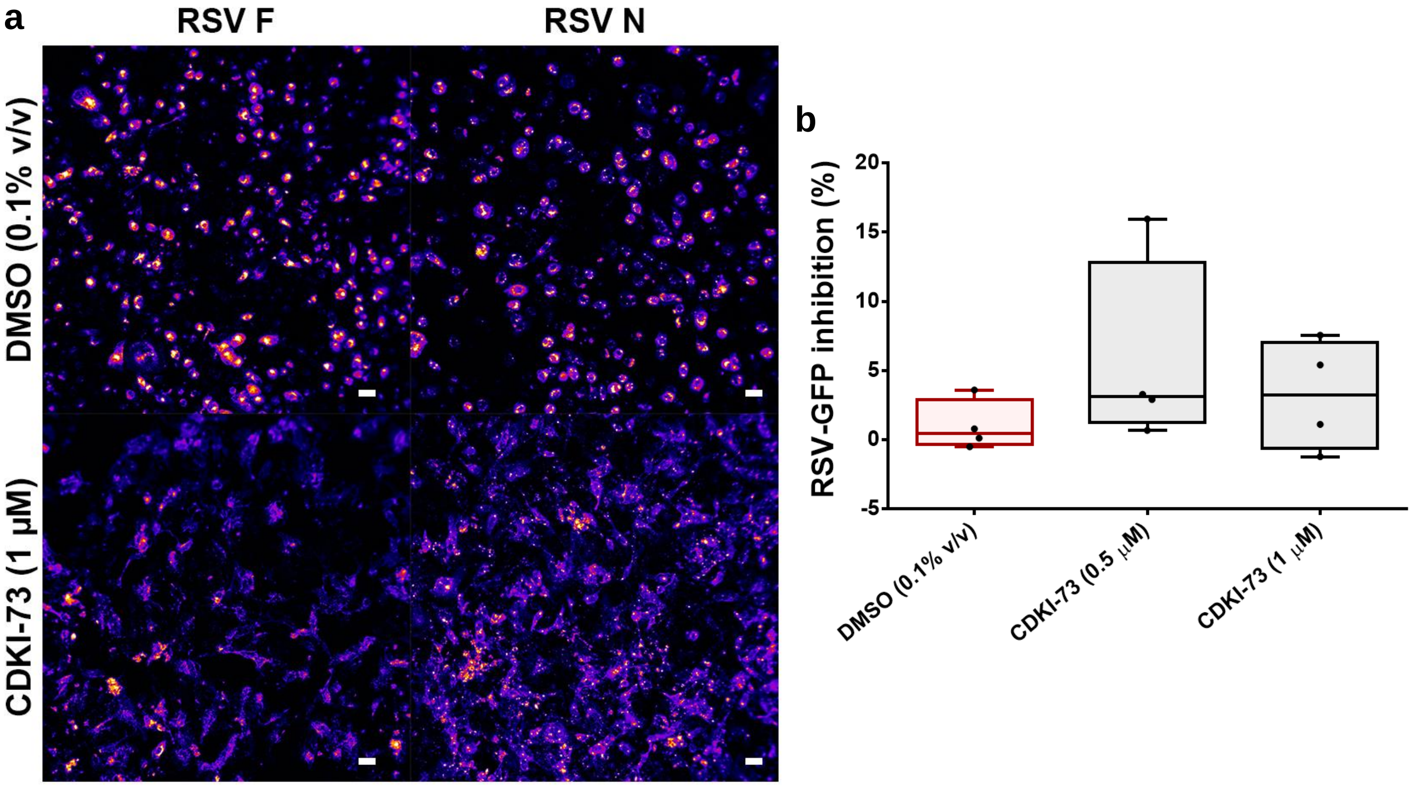

Supplementary Figure 4. Inhibition of Rab11-mediated trafficking causes redistribution of viral proteins but does not reduce RSV-GFP infection rate. HEp-2 cells were infected with RSV-GFP (MOI 0.5) and treated with CDKI-73, an inhibitor of Rab11-mediated trafficking. **(a)** Visualization of F and N immunofluorescence 48 h p.i. Rab11 inhibition causes subcellular redistribution of both viral proteins, relative to cells treated with DMSO control (0.1% v/v). Scale bars = 100 µm. **(b)** RSV-GFP infection rate was quantified 48 h p.i. Rab11 inhibition by CDKI-73 (0.5 µM and 1.0 µM) did not cause a significant reduction in RSV-GFP infection of HEp-2 cells (*n* = 4 independent measurements). Box plots indicate the complete range (whiskers), interquartile range (box), and median (line) of measurements. Statistics were calculated by a one-way ANOVA with a Dunnett's multiple comparisons post-hoc test.

Supplementary Figure 5. Uncropped Western blots.

Supplementary Figure 2

RSV protein expression + corresponding GAPDH (37 kDa) blots to the right

+Statins **+Rho GTPase inhibitors**

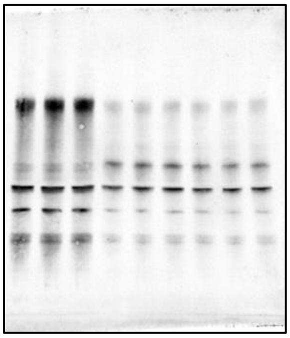
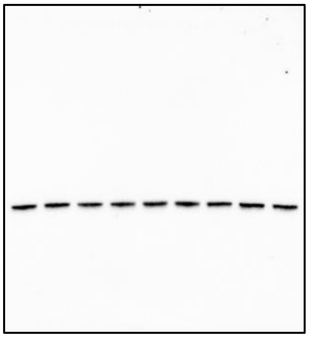
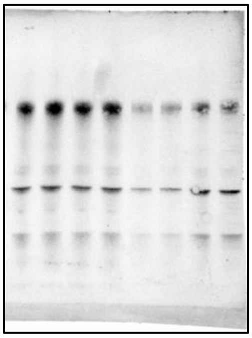
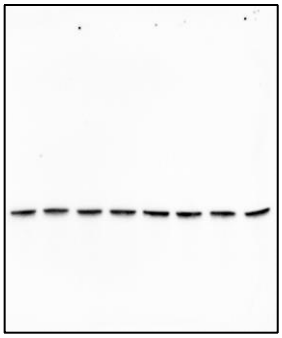

Figure 6b

Total prenylation (DMSO 0.1% v/v)

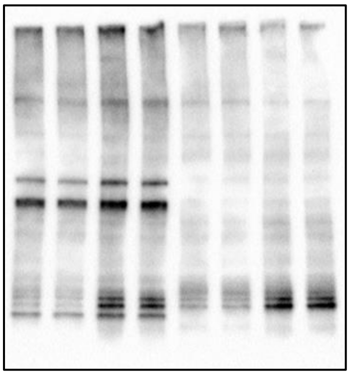

GAPDH (37 kDa)

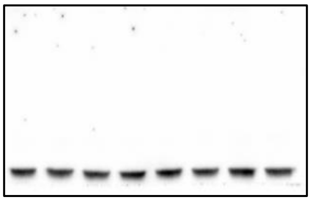

Total Rac1 (21 kDa)

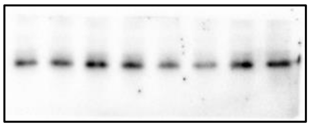

Total prenylation (Atorvastatin 3.5 μM)

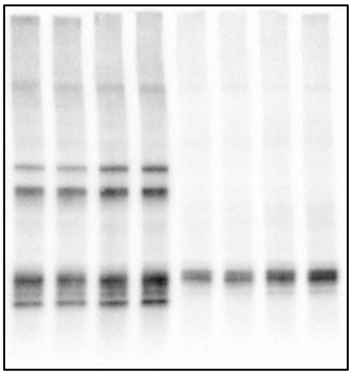

GAPDH (37 kDa)

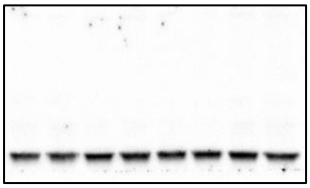

Total Rac1 (21 kDa)

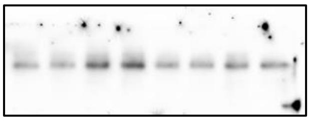

Figure 6c

Active Rac1 (21 kDa)

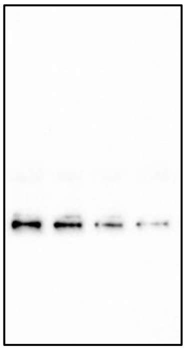

Active RhoA (22 kDa)

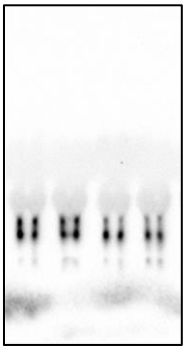

GAPDH (37 kDa)

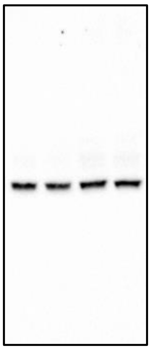

Supplement: Supplementary file 2 — Supplementary Information [file 42003_2021_2754_MOESM2_ESM.pdf]
