# Supplementary material for: Statin-mediated disruption of Rho GTPase prenylation and activity inhibits respiratory syncytial virus infection
Source: Commun Biol. 2021 Oct 29;4:1239. doi: 10.1038/s42003-021-02754-2 (PMC8556396; doi:10.1038/s42003-021-02754-2)
Supplement: Supplementary file 3 — Description of Additional Supplementary Files [file 42003_2021_2754_MOESM3_ESM.pdf]

Description of Additional Supplementary Files

File name: Supplementary Data 1

Description: All data underlying the manuscript figures have been uploaded as a single Microsoft Excel file with captions corresponding to each figure/data set included in the file.
