# Supplementary material for: Statin-mediated disruption of Rho GTPase prenylation and activity inhibits respiratory syncytial virus infection
Source: Commun Biol. 2021 Oct 29;4:1239. doi: 10.1038/s42003-021-02754-2 (PMC8556396; doi:10.1038/s42003-021-02754-2)
Supplement: Supplementary file 5 — Reporting Summary [file 42003_2021_2754_MOESM5_ESM.pdf]

nature research

Corresponding author(s): Maynes, Jason

Last updated by author(s): Oct 3, 2021

Reporting Summary

Nature Research wishes to improve the reproducibility of the work that we publish. This form provides structure for consistency and transparency in reporting. For further information on Nature Research policies, see our Editorial Policies and the Editorial Policy Checklist.

Statistics

For all statistical analyses, confirm that the following items are present in the figure legend, table legend, main text, or Methods section.

n/a	Confirmed	
☐	☒	The exact sample size (*n*) for each experimental group/condition, given as a discrete number and unit of measurement
☐	☒	A statement on whether measurements were taken from distinct samples or whether the same sample was measured repeatedly
☐	☒	The statistical test(s) used AND whether they are one- or two-sided<br>*Only common tests should be described solely by name; describe more complex techniques in the Methods section.*
☒	☐	A description of all covariates tested
☐	☒	A description of any assumptions or corrections, such as tests of normality and adjustment for multiple comparisons
☐	☒	A full description of the statistical parameters including central tendency (e.g. means) or other basic estimates (e.g. regression coefficient) AND variation (e.g. standard deviation) or associated estimates of uncertainty (e.g. confidence intervals)
☐	☒	For null hypothesis testing, the test statistic (e.g. *F*, *t*, *r*) with confidence intervals, effect sizes, degrees of freedom and *P* value noted<br>*Give P values as exact values whenever suitable.*
☒	☐	For Bayesian analysis, information on the choice of priors and Markov chain Monte Carlo settings
☒	☐	For hierarchical and complex designs, identification of the appropriate level for tests and full reporting of outcomes
☒	☐	Estimates of effect sizes (e.g. Cohen's *d*, Pearson's *r*), indicating how they were calculated

Our web collection on statistics for biologists contains articles on many of the points above.

Software and code

Policy information about availability of computer code

Data collection	Drug screening data and fluorescent images used to quantify RSV F and N were collected using the Cellomics ArrayScan VTI software. Immunofluorescent images acquired by confocal microscopy were collected using Volocity v6.3.
Data analysis	Automated image analysis was performed in CellProfiler v2.2.0 and ImageJ v1.52b. Protein targets associated with drug screening hits were identified using DrugBank and queried in PANTHER v15.0. Visual interactome analysis of GO biological processes and molecular components was performed in Cytoscape v3.7.2 using the ClueGO v2.5.5 plugin. Quantification of Western blots by densitometry was performed in ImageJ v1.52b. GraphPad Prism v6.0 was used for all statistical analyses.

For manuscripts utilizing custom algorithms or software that are central to the research but not yet described in published literature, software must be made available to editors and reviewers. We strongly encourage code deposition in a community repository (e.g. GitHub). See the Nature Research guidelines for submitting code & software for further information.

Data

Policy information about availability of data

All manuscripts must include a data availability statement. This statement should provide the following information, where applicable:

- Accession codes, unique identifiers, or web links for publicly available datasets
- A list of figures that have associated raw data
- A description of any restrictions on data availability

The authors declare that the data supporting the findings of this study are available within the paper and its supplementary information files. Detailed data for each graph can be found in Supplementary Data 1. Uncropped Western blots can be found in Supplementary Figure 5.

Field-specific reporting

Please select the one below that is the best fit for your research. If you are not sure, read the appropriate sections before making your selection.

[x] Life sciences   [ ] Behavioural & social sciences   [ ] Ecological, evolutionary & environmental sciences

For a reference copy of the document with all sections, see nature.com/documents/nr-reporting-summary-flat.pdf

Life sciences study design

All studies must disclose on these points even when the disclosure is negative.

Sample size	Sample sizes were not predetermined.
Data exclusions	No data was excluded from this study.
Replication	Experiments were performed in a minimum of three replicates, with the exception of the drug screen which was performed in a single replicate for practicality (lead hits were subsequently validated through additional experiments). Data for Western blots was collected from a minimum of two independent cell lysates.
Randomization	No randomization was performed.
Blinding	Investigators were not blinded to results during data analysis.

Reporting for specific materials, systems and methods

We require information from authors about some types of materials, experimental systems and methods used in many studies. Here, indicate whether each material, system or method listed is relevant to your study. If you are not sure if a list item applies to your research, read the appropriate section before selecting a response.

Materials & experimental systems

n/a	Involved in the study	
[ ]	[x]	Antibodies
[ ]	[x]	Eukaryotic cell lines
[x]	[ ]	Palaeontology and archaeology
[x]	[ ]	Animals and other organisms
[x]	[ ]	Human research participants
[x]	[ ]	Clinical data
[x]	[ ]	Dual use research of concern

Methods

n/a	Involved in the study	
[x]	[ ]	ChIP-seq
[x]	[ ]	Flow cytometry
[x]	[ ]	MRI-based neuroimaging

Antibodies

Antibodies used	Mouse anti-RSV F mAb (Bio-Rad Antibodies, RSV3216/B016), mouse anti-RSV N mAb (Bio-Rad Antibodies, RSV3132/B023), mouse anti-RSV F mAb Alexa Fluor 488 conjugate (Millipore Sigma, 133/1H), anti-mouse Fab fragment Alexa Fluor 488 conjugate (Cell Signaling Technology, 4408), anti-mouse Fab fragment Alexa Fluor 647 conjugate (Cell Signaling Technology, 4410), Streptavidin-HRP conjugate (Cytiva, RPN1231), rabbit anti-Rho pAb (Cell Signaling Technology, 8789), mouse anti-Rac1 mAb (Cell Signaling Technology, 8631), goat anti-RSV pAb (Meridian Bioscience, B65860G).
Validation	All antibodies were independently validated by their respective manufacturers and have been previously cited in the literature.

Eukaryotic cell lines

Policy information about cell lines

Cell line source(s)	HEp-2 cells (ATCC CCL-23), primary human nasal epithelial cells (obtained from healthy volunteers).
Authentication	HEp-2 cells were authenticated by ATCC. PNECs were cultured as per an established protocol (see Methods) and differentiated to a pseudostratified epithelium which was confirmed by microscopy and measurement of transepithelial resistance.
Mycoplasma contamination	All cell lines were negative for mycoplasma contamination.
Commonly misidentified lines (See ICLAC register)	HEp-2 cells were previously described as laryngeal carcinoma cells, and later identified as cervical adenocarcinoma cells. However, these cells are highly permissive to RSV infection and are a common cell model for infection in the literature. We

also validated statin antiviral activity in well-differentiated PNECs which are regarded as the most representative in vitro model of the respiratory epithelium.
